# ACE Gene Variants Rise the Risk of Severe COVID-19 in Patients With Hypertension, Dyslipidemia or Diabetes: A Spanish Pilot Study

**DOI:** 10.3389/fendo.2021.688071

**Published:** 2021-08-19

**Authors:** María Íñiguez, Patricia Pérez-Matute, Pablo Villoslada-Blanco, Emma Recio-Fernandez, Diana Ezquerro-Pérez, Jorge Alba, M. Lourdes Ferreira-Laso, José A. Oteo

**Affiliations:** ^1^Infectious Diseases, Microbiota and Metabolism Unit, Infectious Diseases Department, Center for Biomedical Research of La Rioja (CIBIR), Logroño, Spain; ^2^Infectious Diseases Department, Hospital Universitario San Pedro, Logroño, Spain; ^3^Department of Anesthesiology and Postoperative Care, Hospital Universitario San Pedro, Logroño, Spain

**Keywords:** COVID-19, angiotensin converting enzyme, polymorphisms, hypertension, dyslipidemia, diabetes

## Abstract

Coronavirus disease 19 (COVID-19) caused by the severe acute respiratory syndrome coronavirus 2 (SARS-CoV-2) infection continues to scale and threaten human health and public safety. It is essential to identify those risk factors that lead to a poor prognosis of the disease. A predisposing host genetic background could be one of these factors that explain the interindividual variability to COVID-19 severity. Thus, we have studied whether the rs4341 and rs4343 polymorphisms of the angiotensin converting enzyme (ACE) gene, key regulator of the renin-aldosterone-angiotensin system (RAAS), could explain the different outcomes of 128 COVID-19 patients with diverse degree of severity (33 asymptomatic or mildly symptomatic, 66 hospitalized in the general ward, and 29 admitted to the ICU). We found that G allele of rs4341 and rs4343 was associated with severe COVID-19 in hypertensive patients, independently of gender (*p*<0.05). G-carrier genotypes of both polymorphisms were also associated with higher mortality (*p*< 0.05) and higher severity of COVID-19 in dyslipidemic (*p*<0.05) and type 2 diabetic patients (*p*< 0.01). The association of G alleles with disease severity was adjusted for age, sex, BMI and number of comorbidities, suggesting that both the metabolic comorbidities and the G allele act synergistically on COVID-19 outcome. Although we did not find a direct association between serum ACE levels and COVID-19 severity, we found higher levels of ACE in the serum of patients with the GG genotype of rs4341 and rs4343 (p<0.05), what could explain the higher susceptibility to develop severe forms of the disease in patients with the GG genotype, in addition to hypertension and dyslipidemia. In conclusion, our preliminary study suggests that the G-containing genotypes of rs4341 and rs4343 confer an additional risk of adverse COVID-19 prognosis. Thus, rs4341 and rs4343 polymorphisms of ACE could be predictive markers of severity of COVID-19 in those patients with hypertension, dyslipidemia or diabetes. The knowledge of these genetic data could contribute to precision management of SARS-CoV-2 infected patients when admitted to hospital.

## Introduction

At the time of writing this paper, the COVID-19 has resulted in a pandemic with more than 187 million of confirmed cases around the world that has caused more than 4 million deaths (1) (July 2021). Most of the patients infected by SARS-CoV-2 are asymptomatic or present mild-moderate symptoms. However, in our midst, approximately 10% of patients require hospital admission (2) and 20% of hospitalized patients develop severe respiratory diseases which may quickly progress to respiratory failure, shock and multiorgan dysfunction (3). In hospitalized COVID-19 patients, more than 30% may require intensive care treatment and around 39% of ICU admitted patients die (4). Thus, it is essential to identify those individuals more likely to develop severe forms of the disease for an early precision management. Older age, male sex and pre-existing conditions such as hypertension, obesity, diabetes and chronic kidney disease are risk factors predisposing to serious disease. However, many individuals with these features do not develop severe symptoms, and the causes are not fully understood (3, 5).

The renin-angiotensin-aldosterone system (RAAS), one of the major blood pressure regulatory pathways, could be involved in the pathogenesis of COVID-19, since SARS-CoV-2 uses angiotensin-converting enzyme-2 (ACE2) as the binding receptor to enter the cell. The physiological homeostasis of this system is regulated by the balance of the angiotensin converting enzyme (ACE) and ACE2. ACE converts angiotensin I (Ang I) to angiotensin II (Ang II) which, through its interaction with the angiotensin II type 1 receptor (AT1R), induces a strong vasoconstriction and triggers proinflammatory, proapoptotic and profibrotic pathways in the lung and other organs (6). In RAAS, ACE2 contributes to the inactivation of Ang II, by hydrolyzing it to Ang-1-7, and, therefore, physiologically counters Ang II/AT1R effects, at the same time that stimulates vasodilation and promotes anti-inflammatory, antifibrotic and antithrombotic actions *via* the Ang-1-7/Mas receptor axis (6). In conditions where ACE2 bioavailability is decreased, Ang II-AT1R pathway is potentiated, aggravating COVID-19-induced inflammation and lung injury. Also, high levels and activity of ACE would further increase the activity of the AngII/AT1R pathway. Moreover, advanced age, cardiovascular diseases, hypertension and metabolic diseases such as type 2 diabetes and obesity also lead to further dysfunctions in the renin-angiotensin-aldosterone system (RAAS), likely rendering patients with these underlying comorbidities more susceptible to further repercussions of ACE/ACE2 imbalance (7–9) and references therein].

Genetic polymorphisms in ACE, such as rs4343, rs4341 and the ACE I/D polymorphism, have been shown to affect ACE levels and activity, and confer susceptibility to hypertension (10), type 2 diabetes (11), overweight (12), nephropathy (13, 14) and certain cardiovascular (15, 16) and autoimmune diseases (17). More specifically, the DD genotype of the ACE I/D polymorphism has been associated to higher levels of serum ACE and higher levels of circulating IL6 in patients with myocardial infarction. In contrast, the II genotype has been associated to lower circulating ACE levels, and robust coagulation, and/or enhanced fibrinolysis (7). Since some of these processes have been reported to be involved in the pathogenesis of COVID-19, the DD genotype could predispose to complications of COVID-19 due to higher baseline ACE levels and its consequences (7). Indeed, an association of the DD genotype of the ACE I/D polymorphism with severe COVID-19 has been reported in hypertensive males (18). However, analyzing the I/D polymorphism is laborious and time-consuming and some authors have described a preferential amplification of the D allele (19). Rs4341 and rs4343 polymorphisms are in complete linkage disequilibrium with the ACE I/D polymorphism (19, 20), better reflect circulating ACE levels than direct ACE Alu I/D genotyping (21) and can be easily evaluated by TaqMan assays, therefore they could be better prognostic markers. In the present study we investigated the association of rs4341 and rs4343 polymorphisms of the ACE gene with COVID-19 outcomes in patients with different degree of severity. Identifying genetic variants that influence the severity of COVID-19 could be useful to better understand the physiopathology of the disease or identify new therapeutic targets, through the identification of effector transcripts of the genetic variants that underlie the phenotype. Besides, it could facilitate the early identification of patients genetically susceptible for severe COVID-19 to better monitor them and give them a more appropriate clinical management that may improve their outcomes.

## Material and Methods

### Subjects

In this study, 128 SARS-CoV-2 positive patients (confirmed by PCR from nasopharyngeal swabs) from the first epidemic wave (17 April -29 May 2020), were recruited at the Hospital Universitario San Pedro, Logroño, Spain. These patients were grouped into the following severity of illness categories: i) asymptomatic or mildly symptomatic patients not requiring hospital admission (n=33), ii) severe COVID-19 patients requiring hospitalization in the normal ward (n=66) and iii) critically ill patients admitted to the intensive care unit (ICU) (n=29).

Asymptomatic/mildly symptomatic group was composed by patients with any of the following non-life-threatening symptoms (n=26) such as fever, myalgia, fatigue, rhinorrhea, vomits, sickness, diarrhea, dry cough, cephalalgia, anosmia, ageusia (they had no lung involvement in chest X-ray and had not required oxygen supplements) and also by asymptomatic individuals (n=7) diagnosed with RT-PCR-confirmed SARS-CoV-2 infection (for being a close contact to a COVID-19 positive case). Both types of patients were analyzed in a single group since none of them required hospital admission.

Severe COVID-19 patients hospitalized in the normal ward were patients with symptoms requiring oxygen therapy or patients presenting a high decompensation of their underlying diseases due to the SARS-CoV-2 infection. ICU group were patients that required invasive mechanical ventilation.

Relevant clinical data were obtained from the medical records of patients. Comorbidities were diagnosed by physicians of the Hospital Universitario San Pedro according to the guidelines of different medical Societies. More specifically, the body mass index (BMI) of the patients was calculated as kg/m^2^. Patients were classified by BMI according to the criteria of the World Health Organization (WHO) and the Spanish Society for the Study of Obesity (SEEDO) (22), with obesity defined as a BMI of 30 kg/m^2^ or higher for both sexes. Patients were also classified by blood pressure (BP) values and plasma lipoprotein and triglycerides levels according to the criteria of the European Society of Cardiology, with hypertension defined as office systolic BP values ≥140 mmHg and/or diastolic BP values ≥90 mmHg (23) and dyslipidemia defined as total cholesterol levels ≥250 mg/dl (>200 mg/dl for secondary dyslipidemia or diabetic patients) or low-density lipoprotein-cholesterol (LDL-C) ≥130 mg/dl and/or triglycerides levels ≥200 mg/dl (>150 mg/dl for secondary dyslipidemia or diabetic patients) (24). Similarly, patients were classified according to the guidelines of the WHO and the American Diabetes Association as having diabetes mellitus type 2 if they met one of the following criteria: fasting plasma glucose level of ≥126 mg/dL (≥7.0 mmol/L), 2-hours value of ≥200 mg/dL (≥11.1 mmol/L) in 75 g oral glucose tolerance test (OGTT) or random plasma glucose level of ≥200 mg/dL (≥11.1 mmol/L) (25). Classification of patients with heart conditions (heart failure, coronary artery disease and cardiomyopathies) was performed according to the guidelines of the European Society of Cardiology (26) and diagnosed on the bases of features of systolic or diastolic heart dysfunction in echocardiography, biochemical tests, magnetic resonance imaging at rest, and chest X-ray. Chronic kidney disease was defined according to the Spanish Society of Nephology as albuminuria (ACR ≥ 30 mg/g), urine sediment abnormalities, electrolyte and other abnormalities due to tubular disorders, abnormalities detected by histology, structural abnormalities detected by imaging, history of kidney transplantation, or glomerular filtration rate <60 ml/min/1.73 m^2^. Dementia and/or other neurological conditions category included patients with Alzheimer Disease, senile dementia or Parkinson disease, diagnosed according to the guidelines of the Spanish Society of Neurology (SEN) (27). The criterion for the diagnosis of COPD was defined by a post-bronchodilator FEV1/FVC ratio lower than 0.7 according to the Spanish COPD Guidelines (GesEPOC) (28). The “Others” category included patients with cancer, hepatic chronic disease or peptide ulcer.

Mortality was evaluated as patients’ death within 90 days from SARS-CoV-2 infection detection by PCR (COVID-19 disease onset).

This study was performed following the Helsinki Declaration and was approved by an independent ethical committee for clinical research (*Comité de Ética de Investigación con medicamentos de La Rioja*, CEImLAR, reference number PI-412). All patients or their representatives/relatives gave their consent to participate in the study.

### Genomic DNA Isolation and Genotyping

Genomic DNA was extracted from buffy coat layer using the DNeasy blood and tissue kit (Qiagen, Hilden, Germany). The rs4341 and rs4343 genotyping of the DNA samples was carried out in an Applied Biosciences (ABI) 7300 RT-PCR system using predesigned TaqMan SNP Genotyping Human Assays from ABI (Foster City, CA) according to the manufacturer’s instructions. The ID assays used were C:29403047_10 and C:11942562_20 for rs4341 and rs4343 respectively.

### Circulating ACE Levels Determination

Serum ACE levels were measured by enzyme-linked immunosorbent assay (ELISA) using a commercially available kit from R&D (Human ACE Quantikine ELISA Kit, R&D, Minneapolis, USA).

### Statistical Analysis

Categorical data are presented as counts (percentages) and were analyzed using the χ^2^ test or Fisher’s exact test. Quantitative values are expressed as mean ± standard deviation. Normal distribution of quantitative variables was checked using the Shapiro-Wilk test. Quantitative data were evaluated with the Kruskal-Wallis test followed by the Mann-Whitney U test. *P* values <0.05 were considered statistically significant. Statistical analysis was performed using GraphPad Prism 6 (GraphPad Prism^®^, La Jolla, California, USA).

Allele and genotype frequencies, Hardy-Weinberg equilibrium test, and association of the rs4341 and rs4343 polymorphisms with COVID-19 outcome were analyzed using SNPStats online software (https://snpstats.net/) (29). To reduce the potential confounding effects from age, gender, BMI and number of comorbidities, an adjustment for these factors was performed.

## Results

### Distribution of Demographic and Clinical Characteristics

**Table 1** describes the characteristics and demographic data of SARS-CoV-2 infected patients. Mean age of asymptomatic/mildly symptomatic patients was lower than those with more serious illness (p<0.001). A higher frequency of males was found in both groups of hospitalized patients when compared to asymptomatic/mildly symptomatic patients (p<0.01 Hospital ward *vs* asymptomatic/mildly symptomatic; p<0.001 ICU *vs* asymptomatic/mildly symptomatic). A higher frequency of hypertension (p < 0.001) and dyslipidemia (p < 0.001) was also found in both groups of hospitalized patients when compared to asymptomatic/mildly symptomatic patients, and a similar tendency was observed when examining the frequency of type 2 diabetes mellitus (p<0.01), heart conditions (p<0.05) and dementia or other neurological conditions (p<0.001), although the difference only reached statistical significance for hospitalized patients who did not required ICU admission. A higher proportion of patients with chronic kidney disease was also detected in ICU admitted group when compared to asymptomatic/mildly symptomatic group (p<0.05). Besides, we found a higher prevalence of obesity in the group of patients admitted to the ICU when compared to asymptomatic/mildly symptomatic patients group (p<0.01). The percentage of obese patients in the hospital ward group was also higher than in the asymptomatic/mildly symptomatic group, although the difference was not statistically significant. We did not observe significant differences in the prevalence of chronic obstructive pulmonary disease (COPD) among the 3 groups studied. The mortality rate was higher in patients with severe symptoms compared to patients with mild or no symptoms who did not require hospitalization (p<0.05 Hospital ward *vs* asymptomatic/mildly symptomatic; p<0.001 ICU *vs* asymptomatic/mildly symptomatic). Besides, the number of patients who died within 90 days after COVID-19 onset was significantly superior in the ICU group than in the hospital ward group (p<0.01).

**Table 1:** Clinical and demographic characteristics of COVID-19 patients.

	Asymptomatic or mildly symptomatic N = 33	Hospital Ward N = 66	ICU N = 29	*P*-value
Age (years)	43.58 ± 8.7	70.8 ± 20.4***	65.1 ± 10.8***	**<0.0001**
Male	8 (24.2%)	34 (51.5%)**	22 (75.9%)***	**0.0003**
Hypertension	0 (0%)	30 (45.4%)***	12 (41.4%)***	**<0.0001**
Dyslipidemia	0 (0%)	13 (19.7%)**	7 (24.1%)**	**0.0140**
Type 2 diabetes mellitus	0 (0%)	12 (18.2%)**	3 (10.3%)	**0.0287**
Heart conditions (heart failure, coronary artery disease, cardiomyopathies)	1 (2.9%)	12 (18.2%)*	5 (17.2%)	0.0964
Obesity^a^	3 (9.1%)	14 (22.6%)	11 (39.3%)**	**0.0197**
Chronic kidney disease	0 (0%)	4 (6.06%)	4 (13.8%)*	0.0813
Dementia and/or other neurological conditions	0 (0%)	19 (28.8%)***	3 (10.3%)^ϕ^	**0.0009**
COPD (chronic obstructive pulmonary disease)	0 (0%)	7 (10.61%)	3 (10.3%)	0.1518
Others	1 (2.9%)	5 (7.58%)	3 (10.3%)	0.4974
Deceased	0 (0%)	11 (16.7%)*	9 (47.37%)***^ϕϕ^	**<0.0001**

*p < 0.05, **p < 0.01, ***p < 0.001 vs Asymptomatic/mildly symptomatic cases; ^ϕ^p < 0.05, ^ϕϕ^p < 0.01 vs Hospital ward group.

a Obesity: BMI ≥ 30 kg/m2.

Significant differences appear in bold case.

### Genotype Distribution Among Groups With Different COVID-19 Outcome

The genotypes of both polymorphisms were distributed in concordance with Hardy-Weinberg equilibrium in all groups (for rs4341: asymptomatic/mildly symptomatic *P*=0.16, hospital ward group *P*=1, ICU group *P*=0.72; for rs4343: asymptomatic/mildly symptomatic *P*=0.29, hospital ward group *P*=0.81, ICU group *P*=0.72).

**Figure 1:**
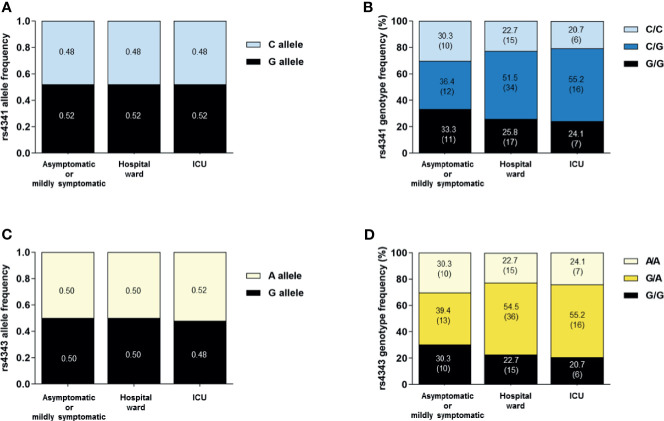
Allele and genotype distribution of the rs4341 and rs4343 polymorphisms among SARS-CoV-2 infected patients. Allele frequencies of rs4341 **(A)** and rs4343 **(C)** polymorphisms. Data are presented as proportions. Genotype frequencies of rs4341 **(B)** and rs4343 **(D)** polymorphisms. Data are presented as percentages. The number of patients is indicated in brackets. All data were adjusted for age and sex.

**Figure 2:**
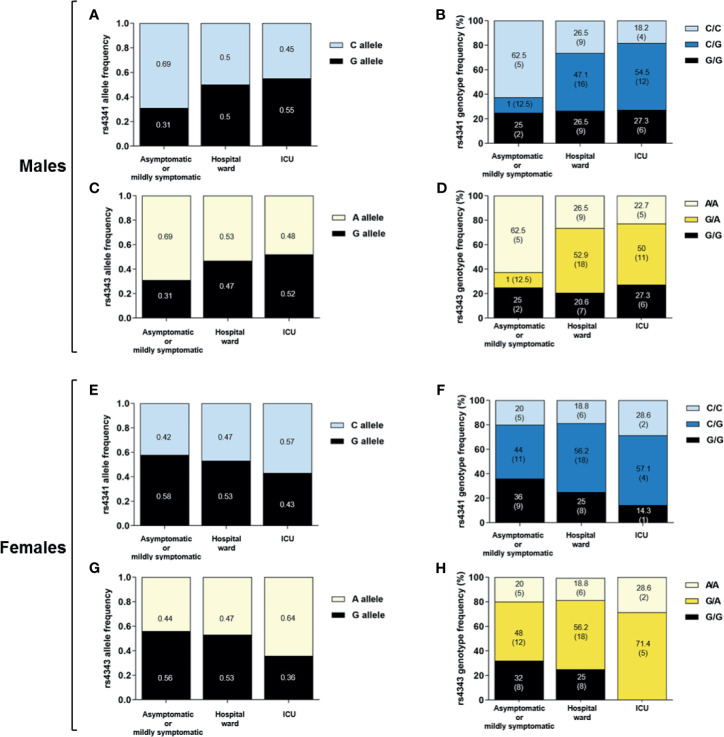
Allele and genotype distribution of the rs4341 and rs4343 polymorphisms among SARS-CoV-2 infected male and female. Allele frequencies of rs4341 and rs4343 polymorphisms among SARS-CoV-2 infected males **(A, C)** and females **(E, G)**. Data are presented as proportions. Genotype frequencies of rs4341 and rs4343 polymorphisms among SARS-CoV-2 infected males **(B, D)** and females **(F, H)**. Data are presented as percentages. The number of patients is indicated in brackets. All data were adjusted for age.

We did not observe significant differences in the genotype and allele frequencies of rs4341 and rs4343 under any of the inheritance models between the 3 groups of SARS-CoV-2 infected patients (**Figure 1**). When we performed the analysis stratifying by sex no significant differences were observed when comparing rs4341 and rs4343 genotypes of male and female patients (**Figure 2**). When evaluating the association of both polymorphisms with the age of the patients and its interaction with the COVID-19 outcome no significant differences were observed either (data not showed).

### Genotype Distribution Among Groups With Different COVID-19 Outcome in Relation to Comorbidities

**Figure 3:**
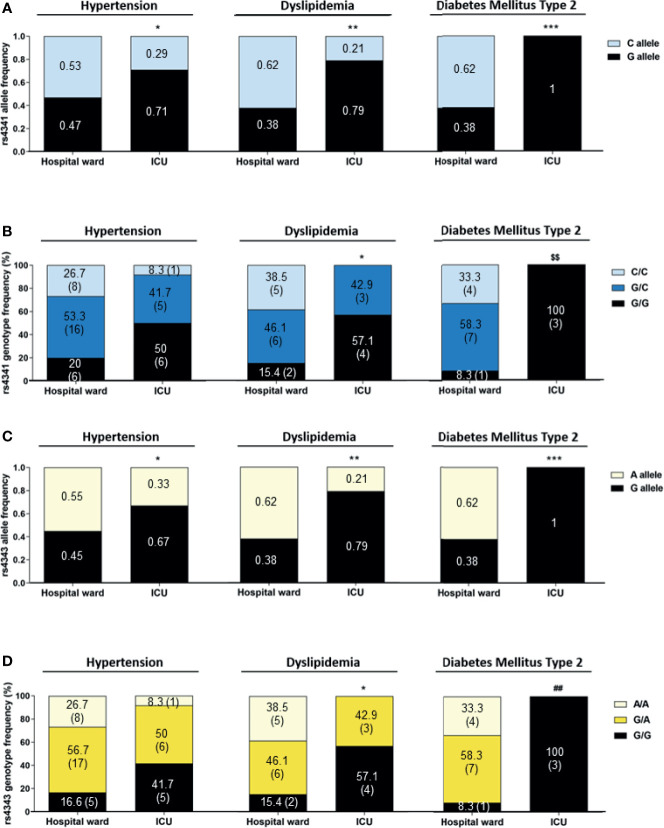
Allelic and genotypic frequencies of rs4341 and rs4343 polymorphisms in COVID-19 patients according to its hypertensive, dyslipidemic or diabetic status. Allele frequencies of rs4341 **(A)** and rs4343 **(C)** polymorphisms. Data are presented as proportions. *p < 0.05, **p < 0.01, ***p < 0.001 *vs* Hospital ward. Genotype frequencies of rs4341 **(B)** and rs4343 **(D)** polymorphisms. Data are presented as percentages. The number of patients is indicated in brackets. *p < 0.05 *vs* Hospital ward group for G carriers (GG+GC *vs* CC and GG+GA *vs* AA). ^$$^ *p* < 0.01 *vs* Hospital ward between GG and GC and CC, GG+GC and CC, GG and GC+CC. ^##^ *p* < 0.01 *vs* Hospital ward between GG and GA and AA, GG+GA and AA, GG and GA+AA. All data were adjusted for age and sex.

The association of the rs4341 and rs4343 polymorphisms with the risk of severe COVID-19 was also evaluated in relation to comorbidities. When we analyzed only hypertensive patients admitted to the ICU, a higher frequency of the G allele in both polymorphisms compared to hypertensive patients hospitalized in the ward was observed (p<0.05) (**Figures 3A, C**). A similar, but not statistically significant tendency was observed in the frequency of the GG genotype (**Figures 3B, D**). When examining the genotypes of dyslipidemic patients divided according to COVID-19 severity, a higher frequency of G allele (p<0.01) and G-containing genotypes (p<0.05) was found in dyslipidemic patients admitted to the ICU than in those admitted to the hospital ward (**Figure 3**). The same differences were observed when evaluating the allele and genotype distribution of rs4341 and rs4343 in diabetic patients (p<0.001 for allele frequencies, p<0.01 for genotype frequencies) (**Figure 3**). These genotype frequencies observed in hypertensive, dyslipidemic and diabetic patients did not differ by sex (data not shown). A higher frequency of GG was not associated to a higher severity in obese patients with SARS-CoV-2 infection (data not shown). No associations were also found among rs4341 and rs4343 genotypes and COVID-19 severity in relation to heart conditions, renal disease, COPD or dementia and/or other neurological conditions (data not shown).

**Figure 4:**
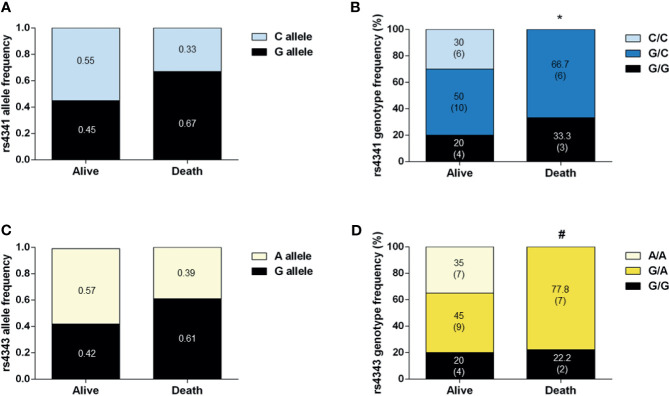
Genotype and allele frequencies of COVID-19 patients in ICU who survived or who died. Allele frequencies of rs4341 **(A)** and rs4343 **(C)** polymorphisms. Data are presented as proportions. Genotype frequencies of rs4341 **(B)** and rs4343 **(D)** polymorphisms. Data are presented as percentages. The number of patients is indicated in brackets. *p < 0.05 *vs* survivors for G carriers (GG+GC *vs* CC). ^#^ *p* < 0.05 *vs* survivors between GG and GA and AA, GG+GA *vs* AA). All data were adjusted for age and sex.

Finally, a higher incidence of G-containing genotypes of both polymorphisms was detected among deceased COVID-19 patients who had been admitted to the ICU compared to survivals (p<0.05) (**Figure 4**).

The G allele of rs4341 and rs4343 polymorphisms is a risk factor for metabolic disorders per se, so to investigate whether the increased risk of severe COVID-19 is due to the potentiation of the metabolic dysfunctions by these alleles, it is independent of the presence of metabolic dysfunctions, or both the comorbidities and the G allele act synergistically, an additional analysis was performed adjusting for age, sex, BMI and number of comorbidities. After adjusting for these additional variables, a significant higher frequency of patients with the GG genotype of rs4341 and rs4343 was found in COVID-19 patients with hypertension (**Table 2**). A higher frequency of the GG genotype was also found in dyslipidemic and diabetic patients even after adjusting for BMI and number of metabolic comorbidities in addition to age and sex (**Table 2**). A higher presence of G-containing genotypes was also found among deceased COVID-19 patients who had been admitted to the ICU after adjusting for the above indicated possible confounding factors (**Table 3**).

**Table 2:** Genotype frequencies of rs4341 and rs4343 polymorphisms in COVID-19 patients according to its hypertensive, dyslipidemic or diabetic status.

rs4341				
Hypertension	G/G	G/C	C/C	*P*-value
Hospital ward	6 (20%)	16 (53.3%)	8 (26.7%)	0.031*
ICU	6 (50%)	5 (41.7%)	1 (8.3%)	**0.03** *^$^*
**Dyslipidemia**	**G/G**	**G/C**	**C/C**	***P*-value**
Hospital ward	2 (15.4%)	6 (46.1%)	5 (38.5%)	0.043*
ICU	4 (57.1%)	3 (42,9%)	0 (0%)	
**Diabetes Mellitus**	**G/G**	**G/C**	**C/C**	***P*-value**
Hospital ward	1 (8.3%)	7 (58.3%)	4 (33.3%)	0.033*
ICU	3 (100%)	0 (0%)	0 (0%)	**0.009** *^$^*
**rs4343**				
**Hypertension**	**G/G**	**G/A**	**A/A**	***P*-value**
Hospital ward	15 (16.6%)	17 (56.7%)	8 (26.7%)	**0.03** *^%^*
ICU	5 (41.7%)	6 (50%)	1 (8.3%)	
**Dyslipidemia**	**G/G**	**G/A**	**A/A**	***P*-value**
Hospital ward	2 (15.4%)	6 (46.1%)	5 (38.5%)	**0.043** ^#^
ICU	4 (57.1%)	3 (42,9%)	0 (0%)	
**Diabetes Mellitus**	**G/G**	**G/A**	**A/A**	***P*-value**
Hospital ward	1 (8.3%)	7 (58.3%)	4 (33.3%)	**0.033** ^#^
ICU	3 (100%)	0 (0%)	0 (0%)	**0.009** *^%^*

Data are presented as number of patients and percentages.

*p-value for group difference between GG and GC and CC.

^$^p-value for group difference between GG+GC and CC.

^#^p-value for group difference between GG and GA and AA.

^%^p-value for group difference between GG+GA and AA.

Significant differences appear in bold case.

All data were adjusted for age, sex, BMI and number of comorbidities.

**Table 3:** ACE genotype frequencies of COVID-19 patients in ICU who survived or who died.

rs4341	Alive	Death	*P*-value
G/G	4 (20%)	3 (33.3%)	**0.021** *^$^*
C/G	10 (50%)	6 (66.7%)	
C/C	6 (30%)	0 (0%)	
**rs4343**	**Alive**	**Death**	***P*-value**
G/G	4 (20%)	2 (22.2%)	**0.041^#^** **0.012** *^%^*
G/A	9 (45%)	7 (77.8%)	
A/A	7 (35%)	0 (0%)	

Data are presented as number of patients and percentages.

^$^p-value for group difference between GG+GC and CC.

^#^p-value for group difference between GG and GA and AA.

^%^p-value for group difference between GG+GA and AA.

Significant differences appear in bold case.

All data were adjusted for age, sex, BMI and number of comorbidities.

### Association of Serum ACE Levels With rs4341 and rs4343 Genotypes

**Figure 5:**
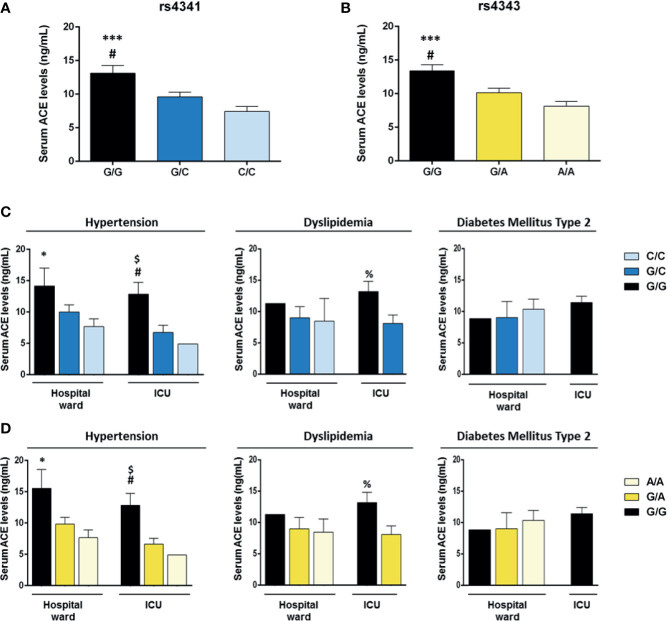
Serum ACE levels of COVID-19 patients according to the genotypes of the rs4341 and rs4343 polymorphisms of ACE. Mean serum ACE levels of COVID-19 patients with different rs4341 **(A)** and rs4343 **(B)** genotypes. ^#^ *p* < 0.05 *vs* G/C group; ***p < 0.001 *vs* C/C group. **(C)** Mean serum ACE levels according to the genotypes of the rs4341 polymorphism in COVID-19 patients with hypertension, dyslipidemia or diabetes hospitalized in the ward or in the ICU. *p < 0.05 *vs* Hospital ward C/C group; ^#^ *p* < 0.05 *vs* ICU G/C group; ^$^ *p* = 0.059 *vs* ICU C/C group; ^%^ *p* = 0.07 *vs* ICU G/C group. **(D)** Mean serum ACE levels according to the genotypes of the rs4343 polymorphism in COVID-19 patients with hypertension, dyslipidemia or diabetes hospitalized in the ward or in the ICU. *p < 0.05 *vs* Hospital ward A/A group; ^#^ *p* < 0.05 *vs* ICU G/A group; ^$^ *p* = 0.059 *vs* ICU A/A group, ^%^ *p* = 0.07 *vs* ICU G/A group. Data are presented as mean ± standard deviation.

Serum ACE levels were significantly higher in patients with the rs4341 GG genotype than in individuals with GC (*p*<0.05) or CC genotypes (*p*<0.001), with the CC genotype being the one associated with the lowest levels of ACE (**Figure 5A**). The same result was observed for the rs4343 polymorphism (**Figure 5B**). This higher elevation of ACE levels in individuals with the GG genotype was also confirmed in hypertensive patients (*p*<0.05), although this elevation was not directly associated with the severity of COVID-19 (**Figures 5C, D**). A similar result was found when examining dyslipidemic patients, although the differences did not reach statistical significance (*p*=0.07), probably due to the small sample size of some groups and to the absence of patients with CC/AA genotype in the ICU group (**Figures 5C, D**). No association was found between ACE serum levels and the genotype of the rs4341 and rs4343 polymorphisms in diabetic patients, nor with its severity (**Figures 5C, D**).

## Discussion

Patients with SARS-CoV-2 infection can experience a wide range of clinical manifestations, ranging from no symptoms to critical illness and even death. However, the causes are still unknown. In this context, and although the sample size of the infected people is small, we found that COVID-19 severity was associated to male gender, as previously described (30). Similarly, older age and a higher number of comorbidities was found in both hospitalized groups compared to asymptomatic patients, in concordance with the literature (3, 31). However, we did not observe a higher mean age or a higher prevalence of hypertension, dyslipidemia, diabetes mellitus, obesity and cardiovascular disease in patients admitted to the ICU compared to those admitted to the ward, suggesting that other mechanisms/pathways could be involved in the severity of COVID-19 illness. In fact, several studies have suggested that variabilities in the genotype distribution of ACE polymorphisms could explain the variable prevalence and clinical outcomes of COVID-19 among different regions of the world (32–34). Thus, we investigated the association of two ACE polymorphisms involved in diabetes and hypertension (rs4341 and rs4343) (10, 11) with COVID-19 outcome in patients from La Rioja. In our study, we did not find differences for rs4341 and rs4343 frequencies among the three groups of patients analyzed when it was assessed without considering the comorbidities. However, we observed that having G allele in rs4341 and rs4343 worsens the outcome of hypertensive, dyslipidemic, and diabetic COVID-19 patients. Moreover, we found that, unlike hospitalized patients in the ward, all diabetic patients admitted to the ICU had the GG genotype. The number of type 2 diabetic patients in this group was very small (only 3 patients), so this result should be viewed with caution. However, our results clearly revealed that rs4341 and rs4343 polymorphisms of the ACE gene are related to the risk of developing severe COVID-19 (ICU admission) in hypertense, dyslipidemic and diabetic patients. Our study confirms previously reported findings of another ACE polymorphism on COVID-19 patients with hypertension (18), however, it did not corroborate the association with COVID-19 severity found by Gomez et al. (18) and other authors (35) for the ACE I/D polymorphism, regardless of comorbidities. Both studies report higher number of hypertensives in the critically ill group than our study [61% (18) and 73% (35) *vs* 41% in our study], statistically different from the severe group, what could explain the differences observed. Unlike these studies, we also found an additional association of rs4341 and rs4343 with enhanced severity in dyslipidemic and diabetic patients. And interestingly, we further found a higher prevalence of the GG, GC and GA genotypes (all containing the G allele) among deceased ICU-patients, not described so far, confirming the deleterious effect of the rs4341 and rs4343 G allele in COVID-19 outcomes. Precisely, 66% of these patients were hypertensive, thus, the presence of this polymorphism could be involved in the hypertension process *per se* as previously mentioned, but also to be a synergistic risk factor for COVID-19 fatality. It is of great interest to investigate whether the increased risk of severe COVID-19 is due to the potentiation of the metabolic dysfunction by these alleles (acting as synergetic) or just because of such comorbidities. Thus, we performed an additional analysis adjusting for age, sex, BMI and number of comorbidities. Our results show that G alleles remained associated with COVID-19 severity after adjustment for these factors, suggesting that both the metabolic comorbidities and the G allele act synergistically on COVID-19 outcome.

Some studies have reported an association of the GG genotype of rs4343 polymorphism and higher circulating levels and activity of ACE (36, 37), which could explain the higher susceptibility to develop severe forms of the disease in patients with the GG genotype, in addition to hypertension and dyslipidemia. SARS-CoV-2 sequesters ACE2 to invade cells, decreasing the bioavailability of ACE2 which entails a reduction in the degradation of Ang II and an exacerbation of the damaging effects of Ang II (38). Thus, the lung injury and inflammation caused by the reduced ACE2 levels due to the viral infection, and also by the hypertension, the dyslipidemia and diabetes, may be worsened by ACE genotypes that further increase ACE levels, and hence Ang II levels, such as the GG genotype of rs4341 and rs4343 herein analyzed. In our study we confirmed that ACE levels are associated with the GG genotype of both polymorphisms, which in turn is associated with greater severity of the disease in hypertensive and dyslipidemic patients. Although according to our data we could not affirm a direct association with COVID-19 severity.

There are obviously several limitations in our study, such as the small size of our cohort. Thus, studies with higher number of patients could be of interest to clarify these results. Large-scale GWAS (Genome Wide Association Study) combined with WGS (whole genome sequencing) studies, such as the one being carried out by the SCOURGE consortium (39), will be very interesting to corroborate these results and to explore if they are extrapolable to the whole Spanish cohort or, in contrast, it is specific to our region.

Our pilot results suggest that evaluating the rs4341 and rs4343 genotypes could be a new diagnostic approach to the clinical management of severe COVID-19 risk of SARS-CoV-2 infected patients with hypertension and/or dyslipidemia and diabetes, that can be easily and quickly analyzed. Although knowing the genotype of these ACE variants does not directly modify the clinical course of these patients, identifying those most at risk of severe COVID-19 will allow better monitoring of these patients to apply all available means to improve their prognosis.

In conclusion, our pilot study suggests that the G-containing genotypes of rs4341 and rs4343 confer an additional risk factor of developing severe forms of COVID-19 in patients with hypertension, dyslipidemia, or possibly with diabetes independently of gender. These genotypes seem to be also associated with an increased risk of death, mainly in hypertensive patients. Thus, the genotyping of rs4341 and rs4343 in COVID-19 patients could facilitate a more appropriate clinical management at admission.

## Data Availability Statement

The raw data supporting the conclusions of this article will be made available by the authors, without undue reservation.

## Ethics Statement

The studies involving human participants were reviewed and approved by Comité de Ética de Investigación con medicamentos de La Rioja, CEImLAR, reference number PI-412. The patients/participants provided their written informed consent to participate in this study.

## Author Contributions

MÍ, PP-M, and JO designed the study, interpreted the data and wrote the manuscript. MÍ, PV-B, ER-F, and PP-M performed the experiments. DE-P, JA and MF-L participated in patient and sample recruitment. All authors contributed to the article and approved the submitted version.

## Conflict of Interest

The authors declare that the research was conducted in the absence of any commercial or financial relationships that could be construed as a potential conflict of interest

## Publisher’s Note

All claims expressed in this article are solely those of the authors and do not necessarily represent those of their affiliated organizations, or those of the publisher, the editors and the reviewers. Any product that may be evaluated in this article, or claim that may be made by its manufacturer, is not guaranteed or endorsed by the publisher.

## Abbreviations

COVID-19, coronavirus disease 19; SARS-CoV-2, severe acute respiratory syndrome coronavirus 2; ACE, angiotensin converting enzyme; RAAS, renin-aldosterone-angiotensin system; ICU, intensive care unit; ACE2, angiotensinconverting enzyme-2; Ang I, angiotensin I; Ang II, angiotensin II; AT1R, angiotensin II type 1 receptor.
