# Corrigendum: ACE Gene Variants Rise the Risk of Severe COVID-19 in Patients With Hypertension, Dyslipidemia or Diabetes: A Spanish Pilot Study

**DOI:** 10.3389/fendo.2021.771445

**Published:** 2021-09-20

**Authors:** María Íñiguez, Patricia Pérez-Matute, Pablo Villoslada-Blanco, Emma Recio-Fernandez, Diana Ezquerro-Pérez, Jorge Alba, M. Lourdes Ferreira-Laso, José A. Oteo

**Affiliations:** ^1^Infectious Diseases, Microbiota and Metabolism Unit, Infectious Diseases Department, Center for Biomedical Research of La Rioja (CIBIR), Logroño, Spain; ^2^Infectious Diseases Department, Hospital Universitario San Pedro, Logroño, Spain; ^3^Department of Anesthesiology and Postoperative Care, Hospital Universitario San Pedro, Logroño, Spain

**Keywords:** COVID-19, angiotensin converting enzyme, polymorphisms, hypertension, dyslipidemia, diabetes

## Error in Figure/Table

In the original article, there was a mistake in **Table 2** as published. Alleles of rs4343 polymorphism were incorrectly written. The corrected **Table 2** appears below.

**Table 2:** Genotype frequencies of rs4341 and rs4343 polymorphisms in COVID-19 patients according to its hypertensive, dyslipidemic or diabetic status.

rs4341				
**Hypertension**	**G/G**	**G/C**	**C/C**	***P*-value**
Hospital ward	6 (20%)	16 (53.3%)	8 (26.7%)	0.031*
ICU	6 (50%)	5 (41.7%)	1 (8.3%)	**0.03** *^$^*
**Dyslipidemia**	**G/G**	**G/C**	**C/C**	***P*-value**
Hospital ward	2 (15.4%)	6 (46.1%)	5 (38.5%)	0.043*
ICU	4 (57.1%)	3 (42,9%)	0 (0%)	
**Diabetes Mellitus**	**G/G**	**G/C**	**C/C**	***P*-value**
Hospital ward	1 (8.3%)	7 (58.3%)	4 (33.3%)	0.033*
ICU	3 (100%)	0 (0%)	0 (0%)	**0.009** *^$^*
**rs4343**				
**Hypertension**	**G/G**	**G/A**	**A/A**	***P*-value**
Hospital ward	15 (16.6%)	17 (56.7%)	8 (26.7%)	**0.03** *^%^*
ICU	5 (41.7%)	6 (50%)	1 (8.3%)	
**Dyslipidemia**	**G/G**	**G/A**	**A/A**	***P*-value**
Hospital ward	2 (15.4%)	6 (46.1%)	5 (38.5%)	**0.043** ^#^
ICU	4 (57.1%)	3 (42,9%)	0 (0%)	
**Diabetes Mellitus**	**G/G**	**G/A**	**A/A**	***P*-value**
Hospital ward	1 (8.3%)	7 (58.3%)	4 (33.3%)	**0.033** ^#^
ICU	3 (100%)	0 (0%)	0 (0%)	**0.009** *^%^*

Data are presented as number of patients and percentages. *p-value for group difference between GG and GC and CC. ^$^p-value for group difference between GG+GC and CC. ^#^p-value for group difference between GG+GA and AA. ^%^p-value for group difference between GG+GA and AA. Significant differences appear in bold case. All data were adjusted for age, sex, BMI and number of comorbidities.

The authors apologize for this error and state that this does not change the scientific conclusions of the article in any way. The original article has been updated.

## Publisher’s Note

All claims expressed in this article are solely those of the authors and do not necessarily represent those of their affiliated organizations, or those of the publisher, the editors and the reviewers. Any product that may be evaluated in this article, or claim that may be made by its manufacturer, is not guaranteed or endorsed by the publisher.

